# Determination by Relaxation Tests of the Mechanical Properties of Soft Polyacrylamide Gels Made for Mechanobiology Studies

**DOI:** 10.3390/polym13040629

**Published:** 2021-02-20

**Authors:** Daniel Pérez-Calixto, Samuel Amat-Shapiro, Diego Zamarrón-Hernández, Genaro Vázquez-Victorio, Pierre-Henri Puech, Mathieu Hautefeuille

**Affiliations:** 1 Departamento de Física, Facultad de Ciencias, Universidad Nacional Autónoma de México, Mexico City 04510, Mexico; daniel_perez@ciencias.unam.mx (D.P.-C.); samuel_amat@ciencias.unam.mx (S.A.-S.); diego.zamarron@ciencias.unam.mx (D.Z.-H.); genvazquez@ciencias.unam.mx (G.V.-V.); 2 Laboratorio Nacional de Soluciones Biomiméticas para Diagnóstico y Terapia, Universidad Nacional Autónoma de México, Mexico City 04510, Mexico; 3 Posgrado en Ciencia e Ingeniería de Materiales, Universidad Nacional Autónoma de México, Mexico City 04510, Mexico; 4 Adhesion and Inflammation Lab (LAI), Aix Marseille University, LAI UM 61, Inserm, UMR_S 1067, CNRS, UMR 7333, F-13288 Marseille, France; pierre-henri.puech@inserm.fr

**Keywords:** relaxation, dissipation, microindentation, polyacrylamide hydrogels, viscoelasticity

## Abstract

Following the general aim of recapitulating the native mechanical properties of tissues and organs in vitro, the field of materials science and engineering has benefited from recent progress in developing compliant substrates with physical and chemical properties similar to those of biological materials. In particular, in the field of mechanobiology, soft hydrogels can now reproduce the precise range of stiffnesses of healthy and pathological tissues to study the mechanisms behind cell responses to mechanics. However, it was shown that biological tissues are not only elastic but also relax at different timescales. Cells can, indeed, perceive this dissipation and actually need it because it is a critical signal integrated with other signals to define adhesion, spreading and even more complicated functions. The mechanical characterization of hydrogels used in mechanobiology is, however, commonly limited to the elastic stiffness (Young’s modulus) and this value is known to depend greatly on the measurement conditions that are rarely reported in great detail. Here, we report that a simple relaxation test performed under well-defined conditions can provide all the necessary information for characterizing soft materials mechanically, by fitting the dissipation behavior with a generalized Maxwell model (GMM). The simple method was validated using soft polyacrylamide hydrogels and proved to be very useful to readily unveil precise mechanical properties of gels that cells can sense and offer a set of characteristic values that can be compared with what is typically reported from microindentation tests.

## 1. Introduction

Mechanical characterization has become a fundamental tool for understanding the behavior and organization of living systems [1]. In particular, it is known that the mechanical properties of the cellular microenvironment play a relevant role in cell behavior, fate and function [2,3,4]. It has become critical to precisely characterize the mechanical properties of biological materials at different scales: the relative/modulations of mechanics of cells, extracellular matrix, basal membrane, tissues and organs are now considered to be powerful indicators of physiological or pathological conditions. The correct determination of the mechanics of biological materials also helps fabricate compliant biomaterials that offer physical properties recapitulating key native conditions found in biological tissues. Thanks to recent progress in technology, the stiffness of biocompatible materials such as polymers or gels can be tuned via an adequate control of their level of crosslinking. The elastic modulus of polyacrylamide hydrogels made in the laboratory can now lie in the physiological range of the softest tissues, for instance [5]. Gradual, yet controlled, dynamic modulations of mechanical properties can also be engineered in soft gels, as rather simple techniques allow for material stiffening [6], softening [7] or reversible cycling [8] during cell culture, thereby restoring the natural changes occurring during the reconfiguration of a native matrix/tissue during pathological processes or wound healing and regeneration.

To allow all this, the mechanical properties of the biological materials and of their biomimetic counterparts need to be correctly imitated and, therefore, precisely characterized when using model substrates. Indeed, mechanobiology studies have shown that only slight changes in the order of only fractions of kPa of the mechanical moduli of a given substrate may influence cellular behavior greatly [9,10]. Although measuring the stiffness is typically a well established task in the field of materials science and engineering in general, this is challenging for biological materials because they are very soft (in the Pa to kPa range), adherent, wet, porous and generally spatially inhomogeneous [9], besides their temporal stability or even evolution under cell culture conditions [11]. There are several protocols that describe non-destructive mechanical characterization methods for obtaining bulk (tissue level) or local (i.e., at the microscale) information [12]. Microindentation [13] and atomic force microscopy (AFM) [14,15] are the most common, but micropipette indentation techniques [16] as well as magnetic and optical tweezer-based methods [17] were also proposed for performing such indentations in the lower (several pN) range of forces.

When aiming at measuring the mechanical properties of a material, it is important to consider that linear elastic materials can be characterized by two physical constants, their Young’s modulus and Poisson’s ratio. In contrast, the mechanical properties of non-linear elastic materials cannot be represented by constants, but are described by parameters that are functions of the material’s deformation: for example, the non-linear shear modulus and the non-linear stretch modulus [18]. On the other hand, viscoelastic materials present a stress–strain response that changes with the strain rate [18]. This dependence can either be described by the relaxation and creep moduli in the time domain, or by the dynamic storage and loss moduli in the frequency domain. It has to be underlined here that referring to the “stiffness” of a material is probably improper to describe the mechanical properties of cells and tissues. The Young’s modulus (elastic modulus) is only a partial view of the actual complex mechanical characteristics of the biological materials under study. For instance, in 2018 Ben Amar revisited the modeling of tissues’ hyperelasticity and viscoelasticity and demonstrated that biological materials with a rather disordered structure present strong non-linearities that are absent from the polymeric gels used to imitate them under the same conditions [19]. Cell behavior in vitro can also be very much dependent on material dissipation timescales and a precise control of this parameter is now needed [20]. Recently, the use of hydrogels with viscoelastic properties has become very important to increase the fidelity of the culture substrate materials in vitro [4]. These materials, indeed, look more similar to real tissues, are dissipative in nature, and restore native cellular behavior in culture [21]. By experimentally controlling the viscous and elastic phases separately [22], biomaterials also help elucidate complex mechanobiology phenomena and will certainly impact the field further [23], as cells sense and respond to dissipative substrates [24]. Storage and loss moduli must then both be reported when characterizing mechanical properties, as cells respond to both elastic and viscous parts of their naturally viscoelastic substrates modifying their internal structures and nuclear envelope [25]. All this has been demonstrated to be of particular importance in tissue engineering [26], cancer research for treatment [27] or diagnostics [28].

Despite all this, the usually reported stiffness of cells and tissues is typically obtained by very simple experiments and one uses the Young’s (elastic) modulus that is derived from these measurements using an appropriate model. Indeed, this mechanical property is calculated from experimental curves obtained after measuring force/stress when applying a deformation or vice versa. However, when analyzing the stress–strain data and applying the selected model to it, critical information is necessary to guarantee the fidelity of the obtained results [29]. Precise control or at least knowledge of speed/rate, amplitude/depth or angle under which the material is deformed must be reported. Moreover, the adhesiveness of the sample to the probe has to be controlled. Then, the measurements that are collected need to be fed into the appropriate model after verification of its main hypotheses, before derivation of the mechanical properties [14]. Also, the nature of the material under test as well as the technical limitations and conditions of the testing equipment and the environment itself do influence the results and need to be accounted for with precision and reported thoroughly. For instance, in a recent paper it was shown that the Hertz model per se is not correct for the measurements of cells on very soft substrates, calling for a more elaborate model [30]. On a more technical point, the methods for calibration and determination of the contact point in the curves (position when the tip first contacts the material under test) are absolutely critical [31]. Unfortunately, they are too rarely, or hardly, mentioned, and very difficult to compare and port from experiments and labs to other contexts. 

Moreover, due to the very high sensitivity of biological cells to even small changes in mechanics, controlling with very high precision the scale of deformations and forces employed to characterize the mechanics of living matter is paramount. As reported recently, there are high levels of disparity between measurements, either when performed on the same (a priori) material with different methods or with a similar method but in different laboratories [32]. These discrepancies have critical consequences on the understanding of micromechanics and their origin is thought to lie in the lack of consideration for the importance of two critical variables: setting correct and reproducible sample preparation and measurement conditions, and using the appropriate models to calculate the mechanical properties of the samples from the results obtained. In their review, Guimarães et. al. [32] point that the magnitude, directionality and dynamics of the deformations studied during mechanical tests are not sufficiently accounted for when comparing results, for instance. An additional problem comes from the fact that tissue properties are usually derived from analyses performed only at one scale (macro, micro or nanoscale), although results may differ from one report to another, and also from one scale to another. In the case of model, artificial systems, such as soft porous hydrogels, these differences have been reported to arise from experimental manipulations at small length scales and misinterpretations in the models, because the contact area or surface interactions between the tip of the interrogation probe and the sample surface are underestimated. It was shown, for instance, that the true mechanical properties of soft polyacrylamide hydrogels (poroelasticity and viscoelasticity) can be properly accounted for using indentation at small or large scales (indentation depth and tip diameter) by using indentation-relaxation tests and adjusting the duration of the experiment [33].

In this work, we propose a relatively simple method to characterize the time-dependent mechanical properties of biologically relevant materials based on a rapid and localized indentation-relaxation test using a microindenter. We found that when performed under the right conditions at a correct indentation depth and velocity, a relaxation test provides precise and robust information about the material being tested. Because gels’ dissipative properties are critical in mechanobiology as recently shown [20], the relaxation tests also provide more complete information than microindentation about the mechanics of soft gels as perceived by biological cells. This also simplifies the characterization of dissipative soft materials by employing a single force-time test to define the material’s behavior in a broad frequency domain without the need for tedious and complex analysis demanding dynamic tests. Finally, we compared our relaxation-based results with those obtained from microindentation. In addition to providing useful supplementary information on the Supplementary Materials under test, we found that the elastic modulus of soft poroelastic hydrogels measured by microindentation shows a positive correlation with the long *E*(*t* = ∞) and instantaneous *E*(*t* = 0) relaxation modulus and with the storage modulus evaluated at frequencies lower than 1 Hz. Such a relatively simple experiment can be carried out readily at either the beginning or the end of a biological assay and, from it, one can gather important mechanical characteristic data and potentially verify the absence of bias in obtaining mechanical properties of the substrate. This also provides information in both the time and frequency behavior, within ranges that are relevant in mechanobiology [4]. The method is also much more practical and immediate than performing local oscillating indentations or shear stresses. Finally, data processing is fast and transparent and can be easily adapted to serial measurements (a link to our code is provided at the end of this manuscript). 

## 2. Materials and Methods 

### 2.1. Polyacrylamide (PAM) Hydrogels Preparation

Elastic polyacrylamide (PAM) hydrogels were fabricated as reported by Tse and Engler [5]. Briefly, 4% acrylamide and 2% bis-acrylamide stocks [Sigma-Aldrich, St. Louis, MO, USA] were mixed in deionized water (ddH_2_O) and degassed in a desiccator for at least 20 min to fabricate soft PAM hydrogels of *expected* elastic moduli of 1.10 ± 0.34 and 4.47 ± 1.19 kPa [5]. Then, mixtures were polymerized by adding 0.01% (*w*/*v*) of ammonium persulfate (APS) and 0.001% (*v*/*v*) of Tetramethylethylenediamine (TEMED). To achieve a thickness of ~233 µm, 75 µL of the solution were deposited onto 20 mm round glass coverslips which were treated previously with (3-aminopropyl)triethoxysilane (APTES)/glutaraldehyde following [34]; the polymerization reaction was carried out during 20 min at room temperature. Polymerized hydrogels were rinsed with ddH_2_O once and immersed in Dulbecco’s phosphate-buffered saline (DPBS) 1× at 4 °C overnight to swell and equilibrate. 

Additionally, viscoelastic polyacrylamide hydrogels were also fabricated as reported by Charrier et. al. [4]. First, linear polyacrylamide was obtained by polymerization, from a prepared solution of 5% acrylamide plus 0.025% of APS and 0.05% of TEMED in ddH_2_O. The solution was incubated at 37 °C for 2 h to ensure a complete polymerization. Then, mixtures of 4% acrylamide and 2% bis-acrylamide stocks were prepared to fabricate soft viscoelastic PAM hydrogels of *expected* dynamic modulus of *G*′ = 1.6 kPa and *G*″ = 200 Pa respectively, in ddH_2_O [4]. TEMED was added at the acrylamide/bis-acrylamide and linear polyacrylamide mixture and APS was added just before the deposition; mixtures were incubated for 20 min at room temperature. Once polymerized, hydrogels were rinsed with ddH_2_O and immersed in DPBS 1X at 4 °C overnight to swell and equilibrate. It is possible to define the dynamic moduli that will be calculated here by considering that the relation $E=\left(1+2\nu \right)G^{*}$ is true for soft PAM hydrogels [35], with $G^{*}=\frac{G^{\prime }+G^{\prime \prime }}{2}$ and a Poisson ratio of *ν* = 0.457 [36]. Here, the storage and loss shear moduli that were prepared, therefore, should lead to an expected Young’s modulus of the viscoelastic PAM hydrogels of *E* = 1.723 kPa. These gels will be called “soft V-PAM” in the following sections.

### 2.2. Poly-Dimethylsiloxane (PDMS) Preparation

For comparison with a less porous elastic material commonly used in mechanobiology, the common Sylgard 184 commercial form of poly-dimethylsiloxane (PDMS) was used (Dow Corning). Two different proportions of prepolymer:curing agent were employed, namely 10:1 and 15:1 *w*/*w* respectively to obtain two different expected elastic moduli of ~1.3 MPa and ~0.9 MPa, respectively [37]. After mixing the two agents, the mixture was poured on glass slides, degassed and cured at 60 °C for 2 h in a convection oven.

### 2.3. Mechanobiology Test with Fibroblast Culture

Immortalized human fibroblast, namely the BJ cell line obtained from Alejandro Cabrera-Wrooman from the Instituto Nacional de Rehabilitación in Mexico, were cultured in Dulbecco’s Modified Eagle’s Medium (DMEM) high glucose complemented with 10% fetal bovine serum (FBS) and antibiotics penicillin-streptomycin (from Gibco, ThermoFisher Scientific, Waltham, MA, USA) at 37 °C and 5% CO2; 5 × 10^5^ cells were seeded on PAM hydrogel (HG) conjugated with [100 µg/mL] rat tail collagen type I (from Corning Inc, Corning, NY, USA), as described in [38] and after 48 h of culture were fixed with 4% paraformaldehyde in DPBS at 37 °C for 15 min. Cells were permeabilized with 0.1% Triton X-100 and blocked with 10% horse serum. For immunostaining, samples were incubated with a monoclonal antibody against Yes-associated protein (YAP) at a dilution of 1:200 (sc-101199, from Santa Cruz Biotechnology, Dallas, TX, USA). After that, Alexa594-coupled secondary antibody was used for immunodetection (Jackson ImmunoResearch, West Grove, PA, USA). Actin filaments and nuclei were detected by Alexa488-coupled phalloidin and 4′, 6-diamidino-2-phenylindole dihydrochloride (DAPI) staining (from Molecular probes, ThermoFisher Scientific, Waltham, Massachusetts, USA), respectively. Samples were mounted with Mowiol over a rectangular coverslip following [39]. Samples were visualized with an epifluorescence microscope Eclipse Ci-L coupled to a D750 FX digital SLR camera (from Nikon, Tokyo, Japan). Images were captured by using a Plan Fluor 40× objective. The images were quantified, edited and merged by using the open source image processing package Fiji. The cell spreading was measured as the area detected by phalloidin (actin filaments) in isolated cells only. Cell density was measured by counting the number of nuclei (DAPI) covering an area of 547.95 µm × 365.3 µm. Finally, localization of nuclear YAP protein was calculated by measuring fluorescence intensity, using the corrected total cell fluorescence [CTCF = Integrated Density − (Area of selected cell × Mean fluorescence of background readings] [40] in the area merged by DAPI.

### 2.4. Microindentation and Relaxation Tests

Microindentation tests were performed using a commercial microindenter setup, namely the FT-MTA03 Micromechanical Testing and Assembly System (FemtoTools AG, Zúrich, Switzerland). The force vs. displacement data were obtained with a FT-S200 tip (spherical tip with a diameter of 50 µm) with a measurement range of ±200 µN and a maximum resolution of 0.0005 µN (at a low sampling rate of 10 Hz). Measurements were performed with indentation velocities ranging in the 0–100 µm/s, with a sampling frequency of 100 Hz and a 0.2 µm indentation step. Because soft gels were measured, a force limit of 171 µN was set in the experiments. 

Relaxation tests were also performed using the same system, however, in this case, the same probe tip was programmed to stop its vertical course and remain static at a fixed indentation depth for the whole duration of the force measurement (fixed strain measurements). The tests thus consisted of an indentation phase at a predetermined speed (in the range of 10–200 µm/s) down to a position where the force sensor detected a predetermined, user-defined force value (in the range of 2–50 µN). The duration of the measurement (10–600 s) and the frequency of the acquisition of data (1, 10, 100 and 300 Hz) being adjustable parameters which play on the time resolution of our generalized Maxwell model (GMM) spectrum, they were defined accordingly for each sample.

### 2.5. Force Curves Processing

After the tests were performed, collected experimental data were obtained in the form of files containing force and displacement vs. time, that may be easily manipulated and plotted. Those files were then processed using custom-made programs in Python 3.x (Google CoLab, Mountain View, CA, USA) designed to compute the elastic modulus (microindentation tests) as well as the more detailed constitutive mechanical properties (relaxation tests) of the materials under test. From the system, data of force vs. time (FT) and displacement vs. time (DT) could be acquired and plotted to verify the characterization while measuring. All obtained data were then saved and processed to obtain force vs. displacement (FD) curves as well. Before the fitting process, a pre-processing step was implemented to filter and re-sample the data; this represents a critical step for the noisy data typically obtained for very hard materials. Indeed, we observed that the tip tends to present recoiling effects when stopping suddenly or encountering a different local stiffness, which is what happens when the probing tip meets the surface of the sample and just before a relaxation phase begins, respectively. Therefore, the removal of this noise (if present) is needed for finding, with a higher precision, both the tip-surface contact point and the starting point of the relaxation phase, and in some cases, further removal of intrinsic noise during the relaxation phase, are also prerequisites for the model to be able to robustly fit the data. In these cases, filtering was accomplished using a Gaussian filter on the force data where the user has the option to manipulate the width of the Gaussian kernel. 

#### 2.5.1. Determination of the Contact Point

**Figure A1:**
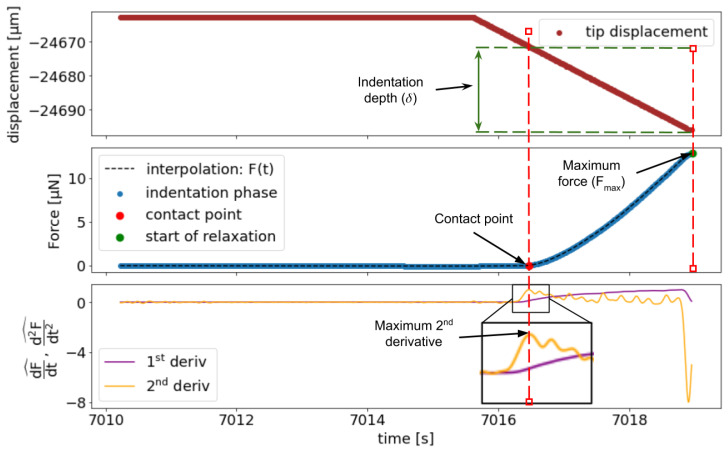
Example of the determination of the contact point, start of relaxation (maximum force) and indentation depth for a relaxation test performed on a 4 kPa PAM hydrogel immersed in ddH2O + 0.1% extran. The first and second derivatives of the force are calculated as a function of time. The superior graph shows the displacement of the probe tip as a function of time. The middle graph shows the time behavior of the measured force. The 1st and 2nd derivatives are calculated from this graph and shown in the inferior graph. Experimental data were interpolated and it was considered that the maximum of the 2nd derivative implied a concavity change associated with the change in force behavior (a linear behavior for the attractive forces domain and a F^2/3^ for indentation forces domain) Thus the position, representative of the contact point is thus precisely determined by the maximum of the second derivative. Here, raw data are shown and time is therefore equivalent to displacement.

It is widely reported that even very small errors in the selection of the contact point may lead to great changes in the resulting elastic modulus, resulting in apparently stiffer materials [41]. In order to process FD and FT curves, we took a great care in identifying the contact point is the first critical part, as it enables the precise determination of the indentation depth, which is mandatory to calculate the mechanical properties of the material under test (see Appendix A and in particular Figure A1). 

#### 2.5.2. Indentation Data Analysis

To fit experimental data obtained from mechanical characterization of materials, the following procedure was used, based on 3 different models typically employed in indentation of soft materials: Hertz model, JKR (Johnson, Kendall and Roberts) and DMT (Derjaguin, Muller, and Toporov) models [14]. Each model has a particular set of hypotheses to be valid and is indeed only appropriate within specific conditions of adhesion between the tip and the indented surface, since the adhesion forces originating at the tip–substrate interface and caused by surface tension or non-specific interactions must be considered in the fitting model for a correct calculation of the material’s mechanical properties [42]. As such, JKR and DMT models are approximations used to describe adhesive contacts during indentation: JKR is useful for contacts on compliant materials with a high surface energy and large contact radius [43] while DMT is employed for contacts on stiff materials with low surface energy and small contact radius [14]. The Tabor parameter *µ* defined in Equation (1) below [44] serves to discriminate which model is the appropriate one to use: if *µ* < 0.1 then the DMT model is used but if *µ* > 5 the JKR model is more correct [45].

(1) $$\mu =\;{\left(R\Delta \gamma^{2}/E_{r}^{2}z_{0}^{3}\right)}^{1/3}$$

To determine *µ*, the tip radius *R*, the adhesion work Δγ, the reduced modulus *E_r_* and the separation distance at equilibrium *z*_0_ (defined in [46] as the range of attraction of adhesive forces, close to atomic distance and ranging in 0.1–0.3 nm) must be determined [42,47]. Here, we used *z*_0_ = 0.3 nm [48] and Δγ was determined using the magnitude of the adhesion force *F_adh_* in the retraction section of the curve of Figure 2D in the following Equation (2):

(2) $$\Delta \gamma =-2F_{adh}/3\pi R$$

Finally, the reduced modulus *E_r_* can be calculated from Equation (3):

(3) $$E_{r}=E/\left(1-\nu^{2}\right)$$

where *ν* is the Poisson ratio, which for acrylamide has been measured to be 0.457, close to 0.5 [36], and *E* is the Young’s modulus obtained from a Hertz model fitting applied to the sample measured in the attraction-free detergent solution (Figure A3A). The data analysis was performed using Wolfram Mathematica 12.1 (Wolfram Research, Oxfordshire, UK).

#### 2.5.3. Relaxation Data Analysis

**Figure 1:**
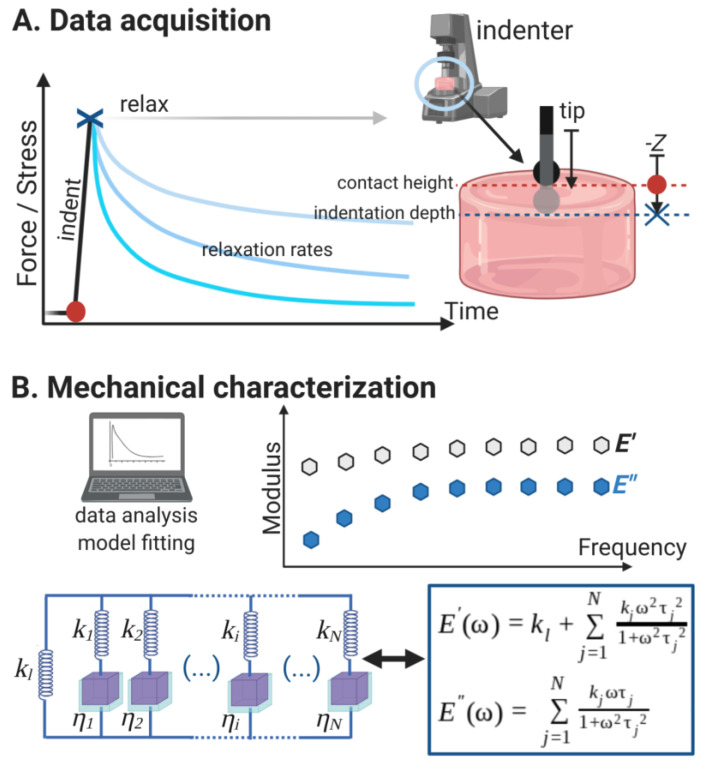
Diagram (not to scale) of the method employed here to study with great precision the mechanical properties and dynamic behavior of polyacrylamide (PAM) hydrogels used in mechanobiology assays. (**A**) Relaxation test performed with a microindenter; it collects all the information necessary to analyze the hydrogels mechanically. The bead is typically 50 µm in diameter and the gel ~200 µm thick. (**B**) The data collected from the relaxation test is adjusted using a generalized Maxwell model (GMM) with the appropriate relaxation times τ_i_ that then allows to obtain the corresponding *k_i_* and *η_i_*. The long term stiffness *k_l_* (or *k_∞_*) is also determined from the model fitting. Finally, the dynamic storage (*E*′) and loss (*E*″) moduli that describe the soft material were calculated, assuming a linear viscoelastic behavior. This complete characterization is more accurate than a microindentation and calculation of the elastic (Young’s) modulus.

For relaxation tests, the contact point also needed to be precisely and objectively determined to set the relaxation start (see Appendix A). Then, the maximum force value *F_max_* was detected to extract its associated time *t_Fmax_* and displacement *d_Fmax_* values, which in turn allowed us to determine the indentation depth δ as *d_Fmax_*-*d_c_* where *d_c_* is the contact distance, as defined by the microindenter. Then, the GMM was used to determine the relaxation modulus *E*(*t*) as follows [49], Equation (4):

(4) $$E\left(t\right)=k_{\infty }+\sum_{1}^{N}k_{i}Exp\left(-t/\tau_{i}\right)$$

where $k_{\infty }$ is the long-term stiffness, $\tau_{i}$ is the *i*-th relaxation time defined as $\tau_{i}=\eta_{i}/k_{i}$ where $\eta_{i}$ and $k_{i}$ are the viscosity and stiffness associated to the time $\tau_{i}$ as represented in Figure 1.

Since the experimental microindenter used in this work measured force instead of stiffness (force per unit area), a form factor was used to transform the obtained parameters to correct for dimensional units. To define this conversion factor, we assumed, following the mode of control set for the microindenter, that the temporal dependence of the relaxation curve was defined entirely by the *k*(t) of the sample and not by the indentation depth δ, that is F(t) = k(t)δ, where F(t) is the force measured by the microindenter. The probing tip indents a surface area and to recover *E* from *k*, a form factor *r* that depends on the surface-tip geometry and the Poisson ratio of the material under test was defined, following [50]. We have *k*(t) = *r E*(t) with *r* defined as follows in Equation (5):

(5) $$r=\frac{4\pi a}{7\left(1+\nu \right)\left(1-\nu \right)}$$

where *a* is the radius of contact defined by $a=\sqrt{R\delta }$, *R* is the tip radius and *ν* the Poisson ratio.

In this particular step, the code that was developed here for the GMM allows the user to define an appropriate number of Maxwell elements (or arms, as depicted in Figure 1B) to be used in the fitting. Once the family of such parameters is defined, it is necessary to scale them using the correct form factor determined above. The plotting of the normalized experimental data together with the obtained GMM fit curve enabled us to verify the accuracy visually but for a less objective decision on the correctness of the fitting, residuals were also calculated (see Appendix B).

From the resulting $\tau_{i}$ and $k_{i}$ that were calculated from the GMM fitting, it is possible to obtain a discrete relaxation spectrum associated with the GMM that defines the mechanics of the material. The *H*(τ) spectrum was constructed for visualization by Dirac delta functions $\delta \left(t-\tau_{i}\right)$ for each relaxation time $\tau_{i}$ with an amplitude $k_{i}/k_{max}$ normalized with regards to the highest stiffness $k_{max}$. This allowed us to compare the relaxation times and dynamic behaviors for different conditions and samples.

Finally, by considering that the PAM hydrogels may present a linear viscoelastic behavior during the relaxation tests, it was possible to predict the dynamic moduli of the material over a range of frequencies. The storage modulus *E*′(ω) and the loss modulus *E*″(ω) were calculated from the $\tau_{i}$ and $k_{i}$ values obtained above, according to Equations (6) and (7) below [49]:

(6) $$E^{\prime }=k_{\infty }+\sum_{i=1}^{N}\left(k_{i}\tau_{i}^{2}\omega^{2}\right)/\left(1+\tau_{i}^{2}\omega^{2}\right)$$

(7) $$E^{\prime \prime }=\sum_{i=1}^{N}\left(k_{i}\tau_{i}\omega \right)/\left(1+\tau_{i}^{2}\omega^{2}\right)$$

Thanks to the estimation of a criterion of cross validation (see Appendix B), it was found that *N* = 3 is sufficient to describe the mechanical properties of soft PAM hydrogels using the GMM, and it allows overfitting of the data to be avoided.

### 2.6. Statistical Analysis

For all the data presented here in every condition, at least 2 samples were characterized independently. Statistical analysis was performed using GraphPad Prism 8 (GraphPad Software, San Diego, CA, USA). Data are consistently presented as mean ± standard deviation. For comparison between different conditions, a variance analysis was achieved (one way analysis of variance, ANOVA) with a Tukey correction for multiple comparisons. Correlation between conditions was quantified using a Pearson correlation.

## 3. Results

The technique (schematically presented in Figure 1A), and the data it produces, enable the determination of the constitutive elements of a GMM where a long-range spring *k_l_* is combined in parallel with a consecutive association of N parallel dashpots and springs (depicted as (*η_i_*; *k_i_*) in Figure 1B). The GMM is advantageous over the simpler viscoelastic linear models, such as the standard linear solid (SLS) model since it considers the non-homogeneous disorder at microscale: the material relaxation occurs according to a time distribution rather than at a single time. This is a definition that falls closer to the mechanics of a substrate as perceived by biological cells, which appear to be able to maximize their spreading if their dissipative timescales match that of the material [20]. Moreover, relaxation tests have been proven to allow the separate quantification of the viscous and poroelastic contributions occurring in soft hydrogels used in mechanobiology [51]. To validate our relaxation test method, we have tested the mechanical properties of soft polyacrylamide (PAM) hydrogels and compared the results with classical indentation tests, either static or using oscillations over a frequency range, as classically used in material characterization (see Appendix C, Figure A8).

### 3.1. Force Curves and Correction for Tip Displacement

**Figure 2:**
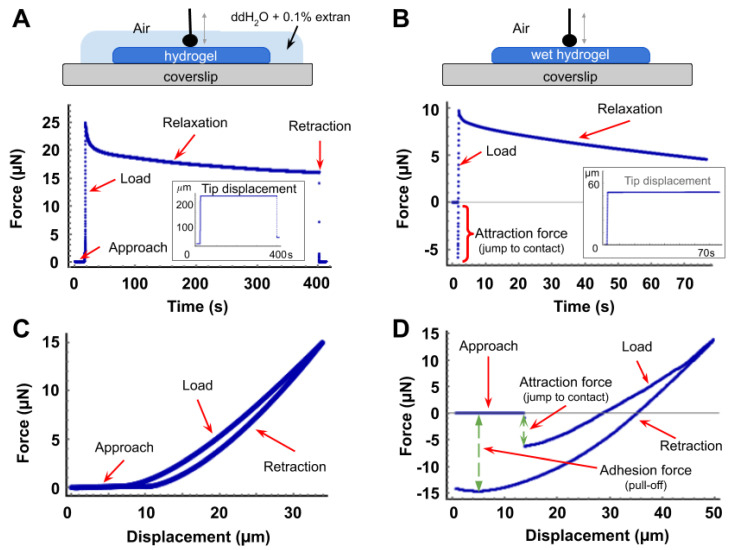
Force curves of microindentation-relaxation assays. Graphs of (**A**,**B**) are force-time curves representative of the relaxation of a soft polyacrylamide hydrogel using a spherical tip with a 25 µm radius. Red arrows signal the different sections of the curve and the embedded insets show the constant position of the tip, equivalent to a constant indentation of the sample. (**A**): relaxation curve obtained from a sample measured when submerged in a detergent solution of ddH_2_O and 0.1% Extran. (**B**): relaxation curve from a measurement in air of a similar hydrogel sample soaked with water (ddH_2_O). Graphs of (**C**,**D**) are force-displacement curves, representative of the microindentation-retraction cycle of a soft hydrogel using the same tip. Red arrows signal the different sections of the curve and the magnitude of the attraction between the tip and the sample. Hysteresis can be observed between load and retraction in both cases. (**C**,**D**) represent the same measurement conditions as in panels (**A**,**B**), respectively.

When analyzing the mechanical data obtained from the microindentation characterization of a soft sample, it is necessary to take several aspects influencing the outcome of the analysis into account. The correct determination of the contact point, the tip displacement and the force of adhesion between the tip and the sample are important parameters to consider. Figure 2 shows representative force curves of typical indentation-relaxation tests carried out on soft hydrogels.

One of the main problems occurring in microindentation is the electrostatic or capillary attraction of the tip by the sample surface, leading to an intermediary complex behaviour between the approach and the contact/loading phase. A reduced adhesion is a prerequisite for mechanical models to be simple and reliable, with as few parameters as possible to input or extract. In order to show how experimental conditions affected our characterization results, two different conditions were tested for the same hydrogel: one hydrogel sample was measured while submerged in a detergent solution of ddH_2_O + 0.1% Extran while an identical soaked sample was measured in air (Figure 2). The role of the detergent solution (Figure 2A,C) was to reduce the surface tension of the liquid and thus avoid the tip–hydrogel attraction that was observed in all the samples moistened with only ddH_2_O water (Figure 2B,D).

Figure 2A,B show force-time (FT) curves where different sections are visible. The first corresponds to the approaching of the tip prior to the physical contact. It is immediately followed by the loading phase (where the force increases to a rate that depends on the material under test), up until the displacement of the tip being stopped by the user at a given force or indentation depth. Then, the relaxation stage begins until the tip is displaced again in the opposite direction, at the same speed, for the retraction section (during which the force decreases at a rate that depends on the material). Therefore, when a constant deformation needs to be imposed on the material, the vertical *z* position of the tip is maintained for as long as the relaxation needs to be evaluated (see embedded inset plots in Figure 2A,B). 

**Figure A2:**
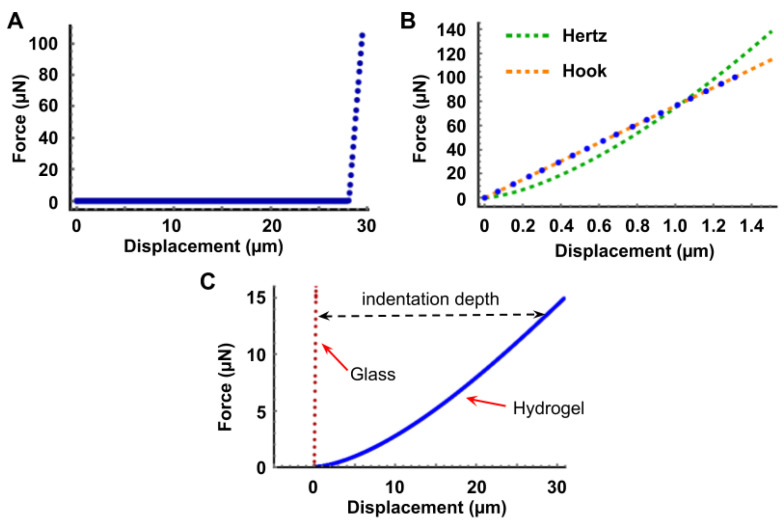
Correction for displacement of the indentation tip. (**A**) FD curve representative of the indentation of glass (indentation of 100 µm/s and a frequency of data acquisition of 100 Hz). (**B**) Hertz model and Hook model fittings of glass indentation data. (**C**) comparison between the FD curve of the indentation of a PAM hydrogel measured immersed in ddH_2_O + 0.1% Extran (blue curve) and the FD curve of the indentation of stiff glass (red), associated to the displacement of the probe tip mechanism. The difference between them is the true indentation depth.

Since the indenting tip is placed manually in the system, it is necessary to rule out or account for any unwanted displacement of the tip mechanism that would impact the measurement, and hence the analysis. To address this, the indentation routine was run at least 20 times and at different indentation speeds (1, 50 and 100 µm/s) on a very stiff glass slide. Since this material presents a stiffness of ~70 GPa [52], it can be considered that within the force sensitivity range of the tip used in the experiments (0–200 µN), there is no indentation of the substrate; hence any measured displacement would correspond to a displacement of the tip mechanism and not of the glass being indented. Figure A2 from Appendix A shows a FD curve obtained from the indentation of a glass slide (panel A) and its corresponding linear fit (panel B) which resulted in a slope of 76.23 ± 0.18 µN/µm (R^2^ = 0.999). Therefore, the difference between the indentation curve of the samples and the indentation curve of the glass is the real indentation depth (as shown in panel C).

### 3.2. Influence of Velocity and Depth of Indentation 

**Figure 3:**
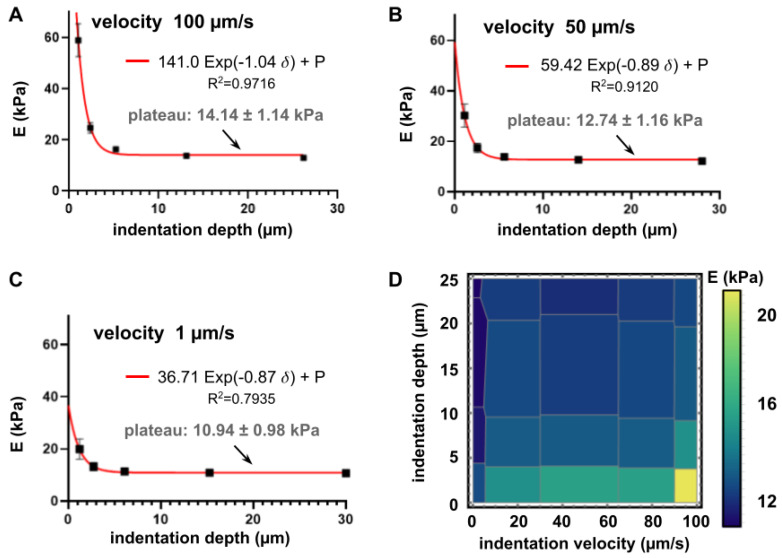
Influence of velocity and depth of indentation on the computed Young’s modulus of a 4 kPa PAM hydrogel, measured in a detergent solution of ddH_2_O + 0.1% extran. Panels (**A**)–(**C**) present the results for velocities of 1, 50 and 100 µm/s respectively. The values presented correspond to calculations using a Hertz model fitting. By increasing the indentation depth, the fitted modulus decreases down to a plateau level of 14.14 ± 1.14 kPa, 12.74 ± 1.74 kPa and 10.94 ± 0.98 kPa, respectively. It was possible to fit those values to exponential decays as a function of the indentation depth, shown in the legend of each graph. In addition to the velocities shown in panels A–C, velocities of 80 and 10 µm/s were tested and shown in the density diagram of panel (**D**) This graph is divided into different Voronoi regions (grey mesh) to visually present the influence of velocity and indentation depth. In panels A–C, error bars represent the mean standard deviation and when not visible, they are smaller than the data point visible dimension.

Once the point of contact has been precisely determined, the influence of experimental conditions like the velocity *v* and depth *d* of indentation are important. Figure 3 summarizes the results obtained when measuring the elastic modulus of a soft PAM hydrogel with an expected value of *E* = 4.47 ± 1.19 kPa. Different indentation depths (~ 1, 3, 7, 13 and 29 µm) and velocities (~ 100, 80, 50, 10 and 1 µm/s) were set during the experiments. It is clearly visible that for all velocities the calculated *E* values decreased with an incrementing indentation depth and rapidly plateaued at a fixed value, which appears to be independent of the depth. An exponential decay fitted very well to the data for each velocity, as shown in Figure 3A–C.

This behavior has already been reported in [53], for AFM indentations of soft agarose gels and lung cells (of similar stiffnesses). In that work, the spherical tips of the pyramidal probes had a 5 µm diameter and the plateau appeared at approximately 200–400 nm indentation inside the materials. Interestingly, the dependence with the indentation depth is similar in both our results and Rico et al. with AFM [53]; apparently for hydrogels the plateau appears consistently at a depth of ~10% of the tip diameter. This seems to indicate an estimate of the depth at which the material behaves as bulky, homogeneous and semi-infinite, which is a strong hypothesis of the models used to determine the Young’s modulus. This depth-dependence of the apparent elastic modulus seen in soft materials like biological cells and gels is not new, yet hardly mentioned in characterization reports. It is very important to determine, since it indicates a minimal (threshold) indentation depth-dependent on the size and geometry of the interrogator tip and the behavior has been recently attributed to surface tension [54]. Remarkably, in our case, the value of the plateau appeared to be also dependent on the velocity of indentation (~20% relative variation for velocity variations between 2 and 5×). As seen in Figure 3 panels A–C and presented in the summary of Figure 3D, the deeper and the slower the indentation of the soft material, the lower its apparent elastic modulus, which is, in a way, an index of its viscoelastic behaviour. To try and discriminate the impact of each parameter in these experiments, a mesh separating the Voronoi regions is presented. It was obtained with Wolfram Mathematica zero order interpolation (Wolfram Research, Oxfordshire, UK), yielding a collection of flat regions, with steps at each data point), each region of the mesh representing the influence of each experimental pair *E = E(v,d)*. 

**Figure A3:**
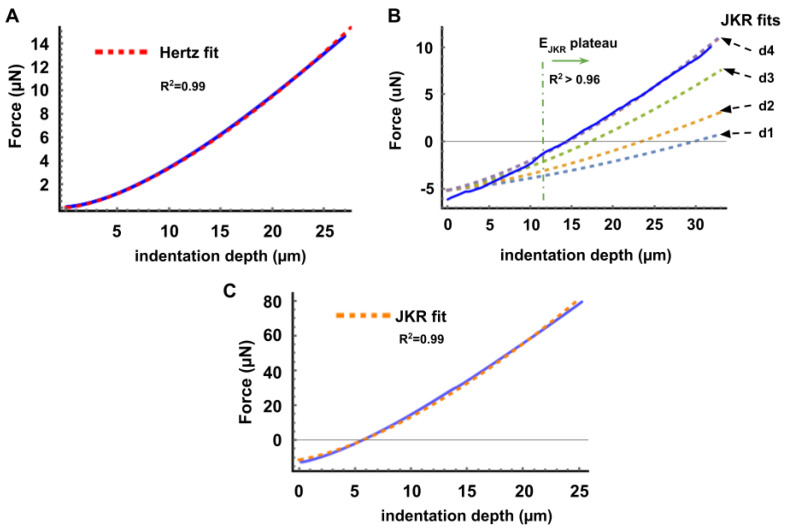
Representative curves of FD fitting processing. (**A**) Hertz model fitting of the indentation of a 4 kPa PAM hydrogel in ddH_2_O + 0.1% Extran. The viscous drag was calculated using the slope of the approach curve (Figure 2C in the main text) obtaining 0.006 ± 0.005 µN/µm at an indentation speed of 100 µm/s, which is negligible compared to the ~0.3 µN change due to relaxation with the same indentation speed. (**B**) JKR model fitting of the indentation of the same hydrogel PAM measured while only soaked. There is an underestimation of the slope (therefore the elastic modulus) when modelling data with distances lower than ~10 µm. Also, for indentation depths greater than ~11 µm the Young’s modulus reaches a plateau value which is similar to the behavior presented in Figure 3 in the main text. (**C**) JKR model fitting of a stiffer hydrogel (~35 kPa, see Table 1) when only soaked in deionized water. There is a clear attraction of ~10 µN, twice that of the softer PAM hydrogel (~5 µN), suggesting a possible dependency of the tip attraction with the stiffness of the sample (acrylamide/bisacrylamide proportions).

Interestingly, our data demonstrate that beyond the minimal threshold indentation depth of approximately 10% (5 µm with a 50 µm–diameter sphere), the computed elastic modulus *E* only depends on the velocity of indentation. This suggests that the stress supported by the hydrogels is strain rate-dependent and this is an intrinsic characteristic of viscoelastic materials. The Hertz model that was used here does not suppose any condition on the velocity of indentation and here we used the average of all plateau values in Equation (3) to derive the reduced modulus and obtain the value of the Tabor parameter. For 4 kPa PAM hydrogels measured while only soaked in air (the condition in Figure 2D) we found a value of the Tabor coefficient of *µ* = 31.36 ± 1.10 × 10^3^ suggesting the use of a JKR model in order to fit the experimental data. However, the JKR shown in Figure A3 panel B clearly shows that this is far from being perfect to accurately fit the data. 

All of this suggests that the mechanical characterization of soft poroelastic hydrogels via microindentation represents a real challenge and that the apparent elastic modulus may still be an incorrect estimator of relative mechanical properties. The hysteresis observed in Figure 2 between the loading and retraction phase (which is a signature of energy dissipation during indentation), as well as the indentation depth-dependency of the value of the elastic modulus clearly indicate that several parameters affect the FD curves, hence the interpretation of the Young’s modulus with the microindentation results. The models may not account for the dissipative phenomena happening in the materials that could be poroelastic and are present in the measurements [55], demanding a more complete characterization method. The relaxation tests described below aimed at improving the easy and reliable characterization in this frame.

### 3.3. Relaxation Tests to Characterize PAM Mechanical Properties

To study the possible viscous origin of the hysteresis found in indentation of soft PAM hydrogels, two other types of hydrogel with distinct, more complex, properties were tested and compared. First, a dissipative viscoelastic PAM hydrogel was fabricated, following [4], see Section 2.1. This V-PAM material is interesting because it offers a crosslinked network PAM with similar elastic properties, but interpenetrated with independent and non-crosslinked linear chains of acrylamide inside the PAM network that are free to move and permit a viscous dissipation. Then, a softer (1 kPa) fully elastic PAM hydrogel was also fabricated, offering a larger pore size in its network than the 4 kPa PAM hydrogels [56]. From relaxation test results, the GMM model can be used to describe the mechanical behavior of soft materials and calculate the storage and loss modulus. To avoid adhesion forces between the tip and the substrate, all samples were characterized while immersed in the attraction-free detergent solution. 

**Figure 4:**
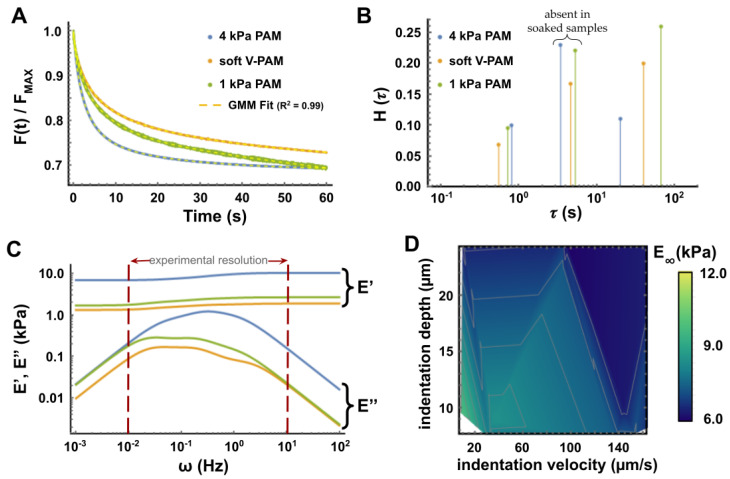
Mechanical characterization of 3 types of PAM hydrogels: 1 kPa elastic PAM, 4 kPa elastic PAM and 4 kPa viscoelastic PAM. (**A**) Comparison of force-time (FT) curves (60 s relaxation indentation at a velocity of ~100 µm/s with a frequency of 100 Hz) with their respective 3rd-order GMM fitting. The curves were normalized to *F_MAX_*. (**B**) Relaxation spectrum obtained from the 3rd-order GMM fitting with Dirac deltas. Distributions of 11 experiments are presented in Appendix A, Figure A4B. (**C**) From the 3rd-order GMM fittings, the dynamic *moduli E′*(ω) and *E″*(ω) were calculated for each sample type. The experimental resolution is the range of frequencies corresponding to the actual measurement times (0–60 s at 100 Hz). (**D**) Influence of the indentation depth and velocity on the long-time stiffness *E_∞_* for the 4 kPa PAM sample. For all graphs, 2 independent samples were measured on at least 5 different locations for each condition.

Figure 4A presents the normalized FT curves showing the relaxation tests performed during 60 s, for the 3 different PAM hydrogels. The system was forced to stop indenting at a predetermined maximum force *F_max_* before relaxation started. We registered the indentation depths for all measurements and they were very similar and varying accordingly with the softest being indented the most: 23.94 ± 1.17 µm for 4 kPa PAM hydrogels, 25.12 ± 3.85µm for the 1 kPa PAM hydrogels and 28.89 ± 3.09 µm for the viscoelastic gels. As expected, the different soft materials presented distinct temporal relaxation responses and the two purely elastic PAM hydrogels relaxed more rapidly than the viscoelastic one. Also, the stiffest (4 kPa PAM) elastic gel stopped relaxing after *t* = 50 sec and settled to a fixed plateau while the other two materials kept relaxing further, although at different rates. The dissipation of force by an elastic material at a constant strain may seem abnormal, but it has been explained by the poroelastic nature of PAM hydrogels: they are made of a porous elastic matrix interpenetrated by an interstitial fluid that can flow and escape, similar to a sponge [33,51,55]. This behavior may explain the hysteresis found in loading–retracting curves of Figure 2 and the difference between the relaxation rates of the two elastic gels thus lies in their porosity difference [56].

**Figure A4:**
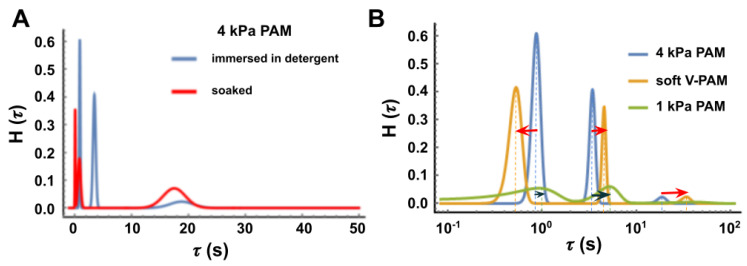
To report on the experimental variability, we plotted a distribution of characteristic relaxation times (represented by Dirac deltas in Figure 4B of the main text), grouping the results of several measurements of different samples. (**A**) Comparison between the relaxation spectra of a 4 kPa PAM hydrogel when measured either immersed in detergent solution or soaked but in air. While the material is identical, the experimental conditions differ and a relaxation time appears at 3.43 ± 0.24 s when the sample is immersed in detergent. Also, an additional relaxation peak appears at *τ* = 0.05 ± 0.05 s in the soaked condition. These differences are probably caused by relaxation effects due to poroelasticity when diffusion happens at different times (see main text). (**B**) Comparison between the different relaxation spectra of samples measured in detergent solution. Each peak represents a normal distribution centered in *τ_i_* with a standard deviation *σ*, obtained by gathering values of *τ_i_* from 2 independent samples for each condition (n = 11). Note: A normal distribution was supposed, with the standard deviation of the data being the deviation of the distribution and the weight is represented by $k_{i}/k_{\infty }$. Because the distributions are gaussian distributions, a smaller peak represents a greater standard deviation, hence a greater dispersion (variability) of the data.

**Figure A5:**
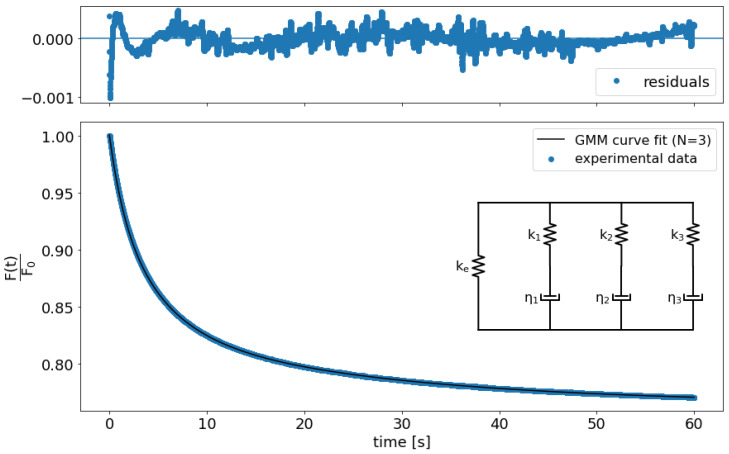
Third order GMM fitting (N = 3) to a relaxation curve of a 4 kPa PAM hydrogel immersed in ddH_2_O + 0.1% Extran. The superior graph shows the residues of the fitting. Experimental data is normalized before adjusting with a GMM fitting.

The temporal behavior of such complex materials as PAM hydrogels offers a better way to describe their mechanics, differentiating them appropriately, unlike the apparent elastic modulus often simply called “stiffness” that makes no difference between 1 kPa and 5 V-PAM gels (see Table 1). The 3rd-order GMM model employed here to describe the relaxation results of Figure 4A unveiled temporal clues behind dissipation in PAM hydrogels. First, it was an excellent fit to the data in the full range of the experimental times, as seen in the figure and confirmed with a cross validation calculation of the residuals (see Appendix B, Figure A5 for a representative FT curve with the residuals). Then, without any consideration for the possible different origins behind the relaxation of soft materials (poroelasticity/liquid phase or network viscoelasticity), the technique proposed here seems to enable a precise determination of the intrinsic and characteristic relaxation times of the materials under test (Figure 4B). Again, the obtained spectra seem to precisely assess the nature of each material very well. Interestingly, the relaxation times accounting for porosity could be those falling in 3–6 s (as shown in Appendix A Figure A4, panel A) because this relaxation time is absent from measurements of soaked samples in air, in which there is no possibility for the medium to be expelled from the structure under pressure. There, the slightly higher relaxation times of 1 kPa and soft V-PAM gels could then be explained by a higher porosity, thus a greater medium outtake effect. In the particular case of the V-PAM hydrogel which is viscous by design, the origin of the relaxation also comes from the presence of linear acrylamide chains delaying or modifying the deformation of the network thus dissipating the energy of its elastic network [4]. The higher relaxation times of the softer and V-PAM gels cause a slower relaxation as observed in Figure 4A. Interestingly, the 1 kPa and soft V-PAM hydrogels also presented very similar elastic behaviors, explained by their similar apparent Young’s modulus. Although only a representative result is shown in Figure 4, a distribution of all the measured curves is presented in Appendix A, Figure A4, panel B. A good consistency is found for the relaxation times, with a broader distribution for the softest elastic gels; however, it is probably caused by a lowest reproducibility of such soft gels or by variabilities caused by swelling, impacting more strongly the softest gels than the others [57]. 

Figure 4C shows the dynamic moduli *E′*(ω) and *E″*(ω) calculated from the parameters derived from the 3rd-order GMM model with Equations (6) and (7). The experimental resolution represented in Figure 4C depicts the range of frequencies that are physically available, corresponding to actual timescales in which the measurements were achieved; it can be recalled that the characteristic times obtained with the GMM offer only a discrete representation of the real spectrum (Figure 4B). The graph of Figure 4C thus confirms that the storage modulus of the three materials represents the actual elastic modulus that was expected from the literature from which we extracted the protocols used for the preparations made in this work (see the compilation of literature with experimental data in Table 1). The loss moduli are also represented and present differences between the three materials, with the V-PAM and 1 kPa elastic PAM being very similar to each other, in the range that is expected from the preparation protocol and reported by others [4]. It is known [55] that the characteristic poroelastic relaxation times of permeable gels are strongly dependent on the experimental conditions and especially the diffusivity. This dependence is in the order of ~$\frac{\sqrt{R_{tip}\delta }}{D}$ where *R_tip_* is the radius of the probe tip, *δ* is the indentation depth and *D* is the diffusivity of the gel (typically ranging in the 10^−10^ m^2^/s) [58]. It can be inferred from the data that the nature of each gel (different porosity and free volume) may explain the variations of the relaxation times found here. This also suggests that the viscosity of the medium will probably influence the mechanics of the gels bathed in it, as found in [59,60]. 

**Figure A8:**
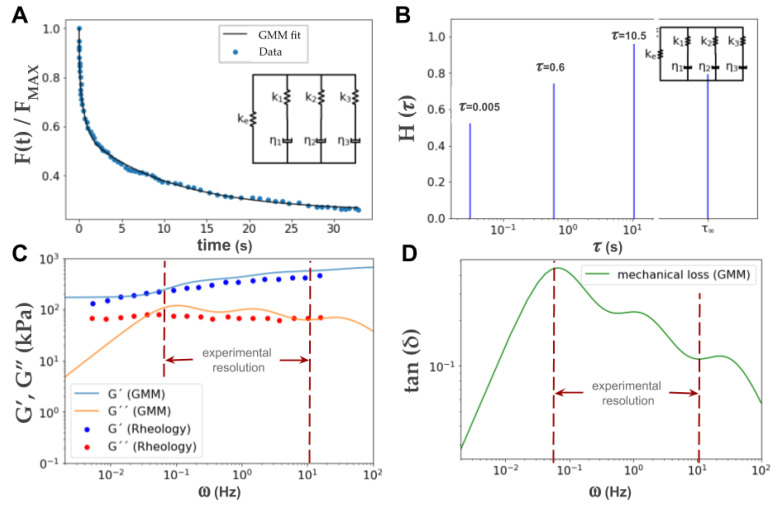
GMM analysis of published experimental data (FT curve) obtained by nanoindentation-relaxation of a biological cell using AFM [61] and comparison of computed frequency behavior with experimental data of dynamic moduli. The data were recovered manually from the published article. This figure corroborates the usefulness of the proposed method to characterize soft materials used in mechanobiology by only using a simple FT curve with relaxation. The raw stress-relaxation data were collected from Figure 1 of reference [61]: a cell was indented and relaxed using an ARROW-TL1 cantilever with an attached 4.7 μm silica bead, and with a spring constant 0.05 N m^−1^. (**A**) Graph reproducing the experimental relaxation data obtained from [61] together with the corresponding GMM 3rd order fit. (**B**) Relaxation spectra associated with the hydrogel sample. We can observe that the relaxation times are of the same order as the ones found in Figure 4 and Figure A4, of our experiments. (**C**) Representation of the dynamic moduli *G*′ and *G*″ obtained from our GMM model (calculated from E* using the relation in Section 2) and compared with the actual experimental data from rheology [61]. From frequencies of ~10^−1^s^−1^, the tendencies are very similar. It is striking that only with the data of the single relaxation curve of panel *A* the full frequency characterization is possible. Interestingly, in [61] the sample was indented 0.4 µm at a velocity of 30 µm/s; the indentation lasted 0.13 s corresponding to the highest frequency (10 Hz) and the relaxation lasted 30 s (~10^−1^ Hz). It was obtained that *G*′(1 Hz) = 432.19 Pa and *G*″(1 Hz) = 98.75 Pa, values that are recommended to be used to compare the results obtained with other characterization methods. (**D**) Losses in elastic energy are defined as tan(δ) = *G*″/*G*′ and it can be observed that the major loss is at frequencies around 10^−1^ Hz and longer times (>10 s) dissipate less elastic energy.

To conclude on the pertinence of our method for obtaining the appropriate frequency behavior of soft materials, we decided to re-analyze published data obtained by another group where both FT curves and discrete frequency results were available [61]. As shown in Appendix C Figure A8, an excellent consistency was found when plotting the extrapolated frequency behavior of the dynamic moduli computed from the FT curve and comparing it to the actual experimental frequency measurements that the authors obtained (in a large range of frequencies). 

Finally, Figure 4D shows the influence of the velocity and depth of indentation on the long-term elastic modulus *E_∞_* of the 4 kPa PAM sample. When compared with the same graph constructed in Figure 3D for data obtained from the Hertz model, it is clear that the variations are smaller for the GMM-derived modulus, around a value of 6 kPa (which has to be compared to the higher ~10 kPa results obtained by indentation and the Hertz model represents at least 50% less variation than that obtained with microindentation). Interestingly, for indentation velocities of ~140 µm/s the calculated long-term stiffness *E_∞_* is almost independent of the indentation depth. This is consistent with previously reported results showing that a higher indentation velocity is preferable, for such experiments [55]. 

### 3.4. Comparison between Microindentation and Relaxation Mechanical Tests

**Figure 5:**
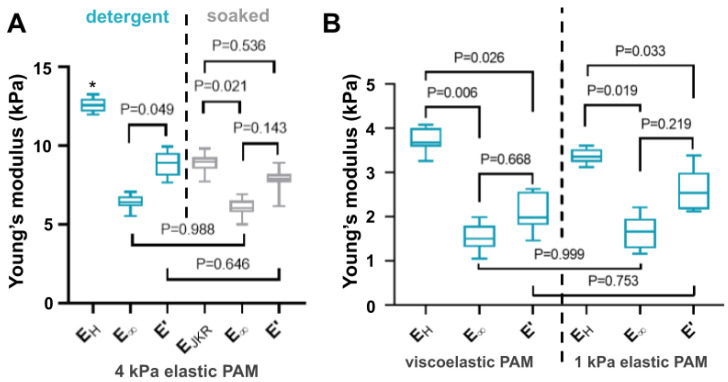
Comparison between the elastic modulus obtained from microindentation and the most relevant model for data fitting, and relaxation tests, using the GMM model. (**A**) Comparison between values calculated for hydrogels immersed in a detergent solution of ddH_2_O + 0.1% Extran (cyan) and only soaked with ddH_2_O and measured in air (grey). In the case of the microindentation test, the Hertz model was used to determine *E_H_* in immersed samples while the JKR (Johnson, Kendall and Roberts) model was preferred to calculate *E_JKR_* for soaked gels. In the case of relaxation tests, the 3rd order GMM model allowed the calculation of long-term elastic modulus *E_∞_* as well as the determination of *E′*(ω) and *E″*(ω) for a given frequency range delimited by 10^−2^–10^1^ Hz. In this graph, *E′*(ω = 1 Hz) is shown and noted E′. (**B**) Same comparison for the soft viscoelastic PAM and 1 kPa elastic PAM. Their apparent elastic moduli when submerged in detergent are very similar when using microindentation (see Table 1), but are much larger than expected (see text). Boxes are the interquartile range (Q1–Q3) and bars extend to the maximum and minimum values. All the experimental data are presented as scatter plots in the Appendix C Figure A9, for more details. A one-way analysis of variance (ANOVA) analysis with Tukey correction was employed for multiple comparisons. It was considered significant statistically with *p* < 0.05, * stands for *p* < 0.01.

When the determination of Young’s modulus of a material is required, for instance for quantifying the influence of substrate mechanics on cell spreading [62,63] or determining forces reliably using traction force microscopy measurements [64], our method also readily enables its determination from the relaxation data and GMM analysis. For that, we compared the calculated values of the elastic modulus obtained with conventional microindentation and with the relaxation tests. The results are shown in Figure 5A for the PAM hydrogels with an expected value of *E* = 4 kPa and in the two configurations of Figure 2 (submerged into a detergent solution and soaked). In the case of microindentation, the Hertz model was used in the attraction- and adhesion-free detergent condition (*E_H_*) while the JKR was preferred for the soaked samples (*E_JKR_*). For relaxation tests, two elastic moduli obtained from the relaxation data and 3rd-order GMM fitting are shown: the long-term elastic modulus *E_∞_* and the storage elastic modulus at 1 Hz *E′*(ω = 1 Hz). A significant difference between the value of the elastic modulus was obtained with the Hertz model between the data of the microindentation performed in detergent and the results computed from relaxation tests. This may be explained by the fact that relaxation tests results are more robust when adhesion effects have to be taken into account. Indeed, Figure 5A shows that there is no significant difference between the instantaneous modulus calculated from measurements of 4 kPa PAM samples either immersed or soaked. Moreover, the use of the relaxation spectra obtained from the GMM fitting enabled a further analysis of differences between the two conditions that helped explain the impact of the indentation velocity as depicted in Figure 3D. Although the relaxation spectra of the 4 kPa PAM samples measured in the two different conditions looked very similar, the samples characterized in detergent solution presented an additional relaxation time $\tau_{i}$ = 3.43 ± 0.24 s which is absent from the soaked samples measured in air (see Appendix A Figure A4A). The GMM viscosity associated with this relaxation time was calculated to be *η_i_* = 5.73 ± 0.67 kPa.s, a value very similar to what was reported for other soft hydrogels under compression [65,66], and could explain the increment of the apparent Young’s modulus when indentation takes place at higher velocities. Indeed, we recall that the instantaneous modulus sums up all the moduli and it only appears in the immersed samples and not in the soaked ones, suggesting that the material viscosity (resistance to flow) may be playing a role in the excess found here. Therefore, when aiming at describing the intrinsic mechanical properties of a material under test, the long-term modulus *E_∞_* is clearly an appropriate characteristic property to use as it appears to be mostly independent of the measurement conditions and is almost not impacted by indentation depth for high indentation velocities (as seen in Figure 4D).

Then, Figure 5B shows the comparison between microindentation and relaxation results for the viscoelastic PAM and the 1 kPa elastic PAM. It is clear that their apparent elastic moduli obtained from microindentation only are very similar (see also Table 1), but are 2 to 3 times larger than expected (1.72 kPa and 1.10 kPa respectively). Relaxation tests, however, provide lower values, much closer to the ones that were expected, as measured by AFM and reported in the literature (Table 1).

All the data obtained from microindentation and relaxation tests are summarized and presented in Table 1. This clearly shows that, in most cases, the Young’s modulus obtained from microindentation (using either Hertz or JKR model depending on adhesion levels observed during measurements) differs from the long-term modulus obtained from relaxation tests and GMM analysis. However, the latter appeared to be the closest to the expected values reported by many groups using AFM measurements. Relaxation tests were observed to be less affected by experimental conditions than microindentation and offered better results. Table 1 also presents the storage modulus *E′* evaluated at a frequency of 1 Hz using the GMM analysis. This value is particularly relevant for materials which are used to study the mechanosensing of biological cells, as explained in [20]. It also offers a better mode of comparison with the dynamic moduli obtained from rheometer characterizations. Also, it has been reported recently that the exact swelling state, which can be difficult to control, reproduce and quantify, plays a very important role in the mechanical properties of PAM hydrogels, especially the softest ones (below 9 kPa): the elastic modulus measured by AFM showed that after only 9h of swelling the elastic modulus of the PAM gels decreased substantially [57].

**Table 1:** Comparison of values of elastic moduli obtained for different methods, materials and conditions: *E* = calculated Young’s modulus, *E_∞_* = long-term stiffness, *E′* = storage modulus evaluated at 1 Hz and *E_ref_* = reported Young’s modulus. Detergent and soaked are the conditions depicted in Figure 2 and dry is a condition for which the sample is not moistened and measured in air at room temperature. The JKR model was used to fit experimental data for soaked and dry samples, and the Hertz model was used for samples immersed detergent solution. All reported values are mean ± standard deviation for each condition (two samples were characterized in at least 6 different locations).

		4kPa PAM (kPa)	4kPa PAM (kPa)	Soft V-PAM (kPa)	1kPa PAM (kPa)	Stiff PAM (kPa)	PDMS 10:1 (MPa)	PDMS 15:1 (MPa)
measurement conditions		soaked	**detergent**	**detergent**	**detergent**	soaked	dry	dry
reported values [reference]	*E_ref_*	4.47 ± 1.19 [5]	-	1.723 ^1^ [22]	1.10 ± 0.34 [5]	34.88 [5]	1.35–2.01 [67]	0.9–1.2 [37]
micro indentation	*E*	8.92 ± 0.55	12.60 ± 0.42	3.73 ± 0.27	3.37 ± 0.17	38.72 ± 8.71	0.91 ± 0.09	0.63 ± 0.01
relaxation tests	*E_∞_*	6.00 ± 0.57	6.41 ± 0.44	1.55 ± 0.31	1.63 ± 0.39	36.01 ± 2.67	1.61 ± 0.48	0.61 ± 0.17
	*E′*	8.25 ± 1.47	8.78 ± 0.80	2.09 ± 0.40	2.57 ± 0.50	39.93 ± 2.54	1.68 ± 0.47	0.65 ± 0.16

^1^ expected E′, see Section 2.1.

Finally, we demonstrated the applicability of the relaxation tests and GMM analysis to stiffer PAM hydrogels and less porous elastomers. The last three columns of Table 1 show the results obtained for the characterization of 40 kPa PAM samples (expected Young’s modulus according to [5]) and two types of MPa-range elastic PDMS slabs. No real difference was observed for these stiffer materials between the results obtained from microindentation and relaxation and the calculated values were in good accordance with the literature. It is thought that those materials are much less porous and thus less sensitive to the hysteresis phenomenon found in soft PAM hydrogels. 

### 3.5. Relevance for Cellular Mechanobiology: Cell Response to PAM Hydrogels of Different Mechanical Properties

In order to demonstrate the relevance of this work for mechanobiology assays, a validation of the PAM hydrogels for cell culture was achieved. We chose immortalized human fibroblasts because they are known to respond strongly to very small changes in the substrate stiffness: they spread more largely on stiffer substrates, which is an easy readout of mechanosensing [68]. A very precise characterization of PAM hydrogels is thus required in this type of mechanobiology assays, within experimental conditions that are as close as possible to the cell culture conditions (immersed in medium) since the chosen substrates have very close rigidities. Moreover, the localization of YAP/TAZ (Yes-associated protein 1 and WW-domain-containing transcription regulator 1) markers and F-actin as well as the change of the size of the cell nuclei were used to visualize a clear difference in the cell mechanoresponse between the two materials. The description provided here only aims at validating the use of our materials in mechanobiology assays and underlying the potential of our precise and robust substrates characterization.

**Figure 6:**
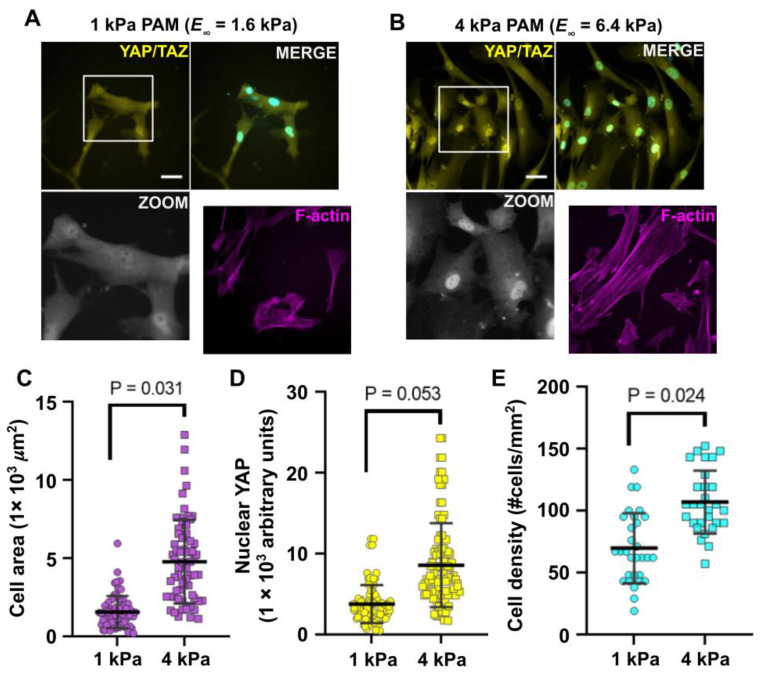
Mechanoresponse of human immortalized fibroblasts on elastic hydrogels with two different apparent stiffness. Human BJ fibroblasts were cultured for 48 h on 1 kPa (panel **A**) and 4 kPa (Panel **B**) polyacrylamide hydrogels functionalized with [100 µg/mL] commercial collagen type I. Subcellular localization of Yes-associated protein (YAP)/TAZ (WW-domain-containing transcription regulator) proteins (yellow) were analyzed by immunofluorescence and epifluorescence microscopy DAPI (4′, 6-diamidino-2-phenylindole dihydrochloride) (cyan) and Alexa488-coupled phalloidin (magenta) were used to stain nuclei and actin filaments (F-actin), respectively. Nuclear localization of YAP/TAZ proteins was highlighted in the zoom squares (gray). (**C**) Cell spreading (cell area) was quantified Figure 1. kPa and 4 kPa substrates. (**D**) Quantification of nuclear localization of YAP/TAZ proteins in fibroblasts cultured on 1 and 4 kPa substrates. Fluorescence intensity was presented in arbitrary units derived from the corrected total cell fluorescence (CTCF), see methods. (**E**) Cell density of fibroblasts cultured on 1 and 4 kPa substrates for 48 h. Data shows that for cells cultured on the stiffer condition there are more cells adhered on the substrates per unit area. Data shown are representative of 3 independent experiments (n = 3). In order to prove statistical differences, an unpaired t-test with Welch’s correction was performed. Bars presented in plots are mean ± standard deviation. Scale bar = 50 µm.

As can be seen from Figure 6, panels A and B, immortalized human fibroblasts presented differences in their behaviour after 48 h of culture on 1 kPa and 4 kPa elastic PAM gels. A first observation was that the cell spreading was significantly greater of the stiffer substrate (panel C). Yes-associated protein (YAP) and transcriptional co-activator with PDZ-binding motif (TAZ) are key effectors of actin polymerization and tensile forces status: the intracellular localization of YAP/TAZ is an important determinant in the regulation of their activity and transduction and it is known to depend on the apparent stiffness sensed by cells. Here, YAP/TAZ proteins showed a stronger nuclear localization (active) in the stiffer material, similar to what is usually reported [69], as shown in panel D (panels A and B, zoomed squares). In addition, the cell density shows a significant difference due to the stiffness of the substrate (panel E). It is also possible to observe that the size of the nuclei of the cells is also greater on the stiffer material and the morphology and size of F-actin fibers are apparently more elongated cells with larger F-actin stress fibers, on the stiffer substrate (panel A vs. panel B). In other experiments, BJ fibroblasts were much more extended with more abundant stress fibers on the 20 kPa PAM hydrogels and on glass, validating a gradual response of such cells on rigidity-tuned substrates (data not shown). 

The point here is that if only microindentation tests results were used to account for the materials stiffness, the two materials would be thought to be 3.4 kPa and 12.6 kPa PAM hydrogels, that is, having very different rigidities (more than 8 kPa apart). We now know, however, that the mechanical properties of the said gels are more complex and the relaxation tests suggest to consider long-term stiffnesses of *E*_∞_ = 1.6 kPa and *E*_∞_ = 6.4 kPa instead, less than a 5 kPa difference. This much smaller stiffness difference between the two gels indicates the large impact on cells of small variations of substrate rigidities, which may occur from one preparation to the other if no particular care is taken in preparing the samples and conserving most stringently the components of gel preparation. These results hence offer a better precision and resolution of the range of the mechanical properties of a material to which the cells respond.

## 4. Discussion

**Figure A9:**
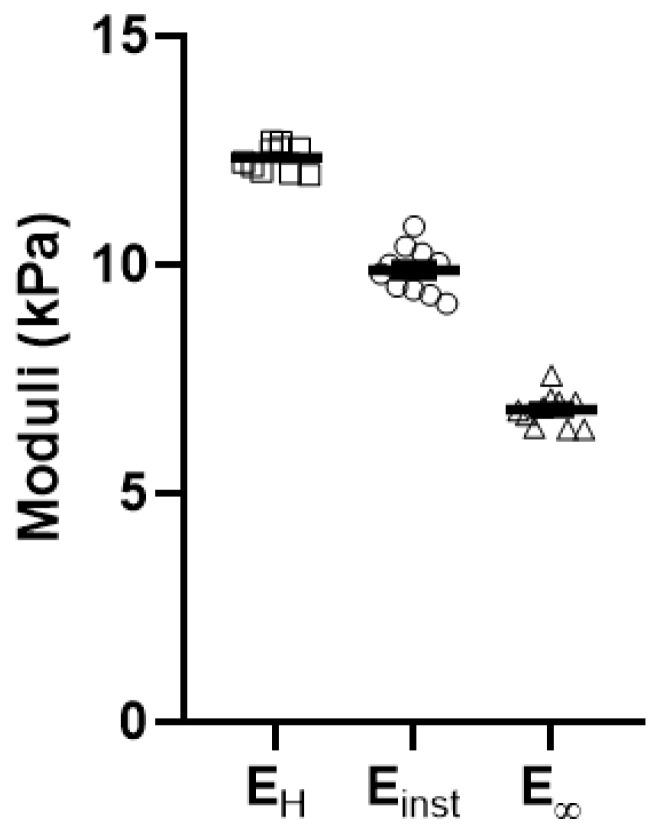
Comparison between the different modules obtained with microindentation (*E_H_*) and relaxation (*E_inst_* and *E_∞_*) for a 4 kPa PAM hydrogel immersed in a ddH_2_O + 0.1% Extran solution.

In this work, we have shown that simple, well controlled, relaxation tests are very useful to characterize soft materials like PAM hydrogels and describe their dynamic behavior in conditions close to those of cell culture. We developed a GMM model and its application to data to extract parameters that are relevant in mechanotransduction studies. We characterized this procedure by designing gels with precise elastic or viscoelastic properties, and obtained results in good agreement with what was expected from literature reports. Careful mechanical characterization tests were performed to compare the values of the actual moduli with the reported ones (see Section 2.1). Consistent values were repeatedly obtained from AFM indentation and microindentation in our laboratories (data not shown). However, slight experimental changes in conditions of preparation and storage, and even small differences in the identification of the contact point, may lead to discrepancies in the determination of the absolute Young’s modulus in the softer gels. For this reason, the gels are called 1 kPa and 4 kPa in this report, using those values as indications only and for simplicity. However, it is important to recall here that the underlying ground of this work is the need to determine the actual mechanical properties of each sample in a rapid and simple manner. This is critical to correctly determine possible slight changes from sample to sample that can explain sometimes subtly different cell responses in mechanobiology. It is also interesting to note that the 4-fold difference between “expected” values of 1 kPa and 4 kPA was conserved in measured values for both microindentation in detergent tests (3.37 kPa and 12.6 kPa) and relaxation tests (1.6 kPa and 6.4 kPa). This effect originates from the strong correlation found between the Young’s modulus *E* and the long-term stiffness *E_∞_* (Pearson correlation of 0.92, see Appendix C Table A1 and Figure A9) but the absolute values differ by less than expected. Biological cells seem very sensitive to slight changes in absolute gel stiffness, calling for very precise measurements of the mechanics sensed by cells, especially when designing biomimetic materials aiming at recapitulating native tissue properties. In addition to the determination of elastic moduli, PAM hydrogels are known to present dissipation and, here, it was possible to quantify it, as shown in the hysteresis curves in Figure 2. This needs to be accounted for in the characterization of materials as recent findings have shown that the relaxation times of culture substrates are critical in mechanobiology, controlling cell spreading dynamics in culture [20]. 

We first determined the correct experimental conditions to perform the desired relaxation tests. Adhesion forces must indeed be avoided to limit possible unwanted interactions biasing the measurements and distorting the final results. Also, a minimal indentation depth threshold of ~10% of the diameter of the tip was identified, similar to what had been reported for AFM in soft materials and even cells [53]. Although much larger spherical tips were used here, interestingly the same ratio was found. Indentation speed was also found to be important here, and this is justified by the fact that PAM hydrogels are poroelastic materials with a liquid phase exiting the material during the indentation compression and diffusing in and out of the surrounding liquid in time [55].

Then, our relaxation assays confirmed that soft PAM hydrogels may dissipate elastic energy with different characteristic times and that this dissipation affects the mechanical characterization by microindentation but cannot be accessed by this only means. Providing more useful information on the mechanical properties of the gels, a GMM was used to describe different materials using their relaxation times with associated stiffnesses and viscosities as depicted in Figure 1B. Dynamic storage and loss moduli were also calculated and found to be accurate in describing the mechanics of the gels in a more general manner than Hertz model or empirical models [55]. Two relaxation mechanisms in soft gels may exist: viscoelastic relaxation due to conformational changes of the polymer chains and fluid-induced poroelastic relaxation. A combination of both is also possible, as seen in soft materials that swell with a solvent. Poroelastic relaxation is considered the dominant relaxation mechanism in PAM gels and it was demonstrated that the mechanical and transport properties of such poroelastic materials can be measured via microscale load relaxation, justifying our approach [33]. We also validated the method for stiffer PAM hydrogels and PDMS, showing that it can be used for other types of material used in mechanobiology.

As a conclusion, when properly performed, the simple relaxation tests proposed here are a very useful tool to describe soft PAM hydrogels, as the results obtained rapidly from this simple, one-step characterization provide a full mechanical characterization of a soft material from a simple FT curve. The practicality and minimal invasiveness of this technique also enables its use at any moment of a biological assay, without perturbing the conditions of the experiment. For comparison with typical microindentation tests, the intrinsic mechanical properties of a material under test may be defined by the long-term modulus *E_∞_*, which is clearly an appropriate characteristic, independent of the measurement conditions and almost not impacted by indentation depth for high indentation velocities. It could then be useful to report long-term modulus *E_∞_*, in decellularized matrices or tissue slides to correctly mimic this mechanical cue in biomimetic polymers. Finally, the relaxation test method, combined with a correct GMM fitting, provides useful information on the dynamic mechanical behavior in a range of frequencies that is relevant in mechanobiology.

## Figures and Tables

**Figure A6:**
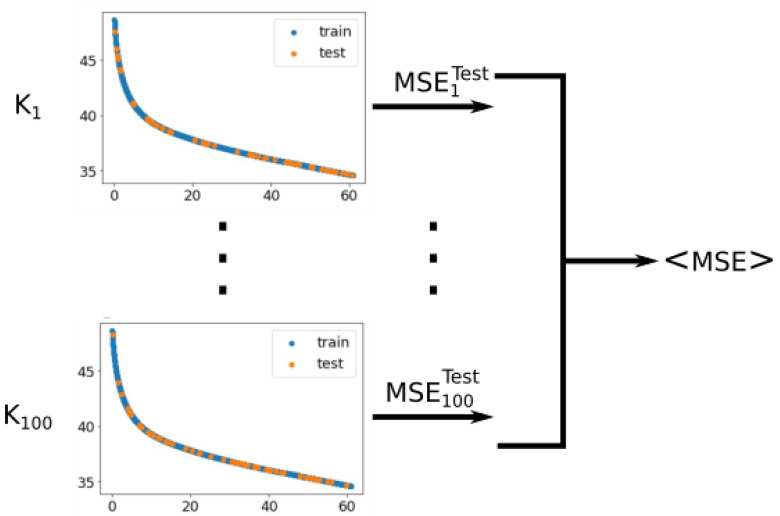
Calculation of MSE as a criterion of cross validation to evaluate the quality of the GMM model fitting.

**Figure A7:**
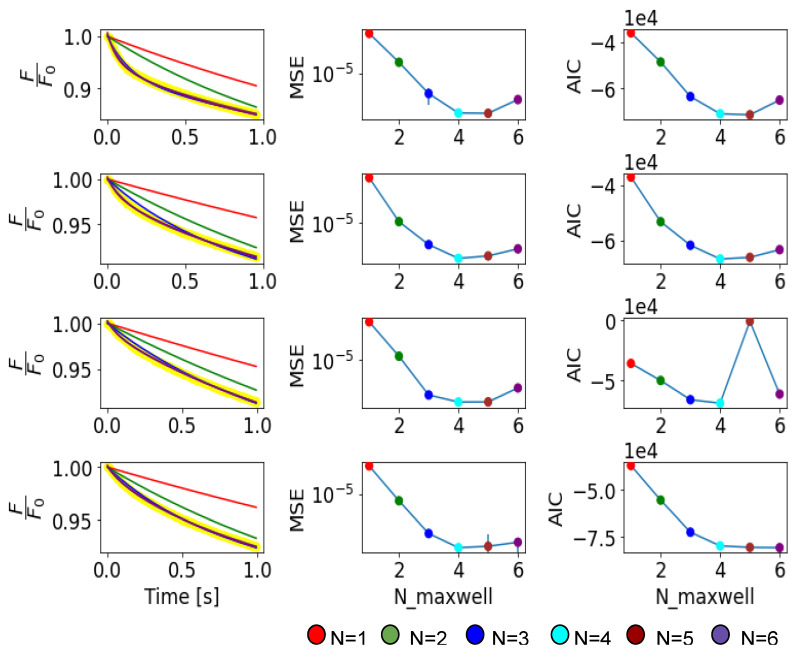
Example of the method of determination of best GMM fitting order for stiff PAM hydrogel samples. The left column shows the experimental data of 4 curves (yellow) together with their GMM fittings of different orders (corresponding to a different number of Maxwell elements, or arms). Each dot presented in the graphs at the center and right columns are the MSE criterion (center) and Akaike information criterion (right) for *N* arms in the GMM fitting, showing which order is the best.

## Data Availability

The data presented in this study are available on request from the corresponding author.

## Supplementary Materials

The Python code written and used in this project for the GMM analy-sis is shared here for use with your own experimental data: https://colab.research.google.com/drive/143jxHL-BaEsIdwxGCFG47H0dKgiUHAJ8#scrollTo=TlOqZSN-Komi. Feel free to comment and propose improvements.

## Appendix A. Notes on FD Curve Processing and Analysis

In our hands, for all samples measured in a ddH_2_O + 0.1% extran solution (to avoid undesired attraction of the tip) the contact point coincides with the position at which indentation begins. However, in the soaked-only samples, it has been reported that the contact point is not necessarily there [14]. We then considered the change of the slope in the FD curve (its first derivative) as a useful way to identify the position of a switch in the force regime. Indeed, when performing indentation tests, the origin of the major contribution of the measured forces is changed from an attraction-led regime in which the force is proportional to the indentation depth *δ* (F_attraction_ ~ *δ*) to an indentation-driven regime in which *F_indentation_* ~ *δ*^3/2^. If these two types of forces are the only ones present during the physical characterization, then the maximum of the second derivative precisely and objectively marks the beginning of indentation (see Figure A1). The variations of the slope in the FD curves are also observed in the FT curves. If $F_{indentation}∼\delta^{3/2}$ and we suppose that $F_{indentation}=F\left(t\right)$ therefore $F\left(t\right)∼\;\delta {\left(t\right)}^{3/2}\;\Rightarrow dF\left(t\right)∼\frac{3}{2}\delta {\left(t\right)}^{1/2}dt\;$and so$\;dF/dt∼\frac{3}{2}\delta^{1/2}$. On the other hand, we have $dF/d\delta ∼\frac{3}{2}\delta^{1/2}$ leading to dF/dδ=dF/dt. This implies that the procedure to find the contact point in the FD curves for microindentation tests is equivalent for the FT curves obtained in relaxation tests. Figure A1 shows the determination of the contact point associated with the maximum of the 2nd derivative with respect to time computed for a relaxation curve for a 4 kPa PAM hydrogel immersed in detergent solution.

## Appendix B. Notes on the Criterion of Cross Validation to Determine a Proper Fitting

This appendix shows the verification of the correctness of the GMM fitting using the visualization of the residues and the subsequent evaluation of a possible overfitting of experimental data.

In order to evaluate the level of acceptability or correctness of the GMM fitting, we used a criterion of cross validation calculating the mean squared error (MSE) defined in Equation (A1):

(A1) $$MSE=\frac{1}{N}\overset{N}{\underset{i=1}{\sum }}{\left(Y_{i}^{test}-{\hat{Y}}_{i}^{test}\right)}^{2}$$

where the $Y_{i}^{test}$ represent the test data and ${\hat{Y}}_{i}^{test}$ the validation data out of a total of *N* data points in *i* partitions. We have:

(A2) $${\hat{Y}}_{i}^{test}=GMM_{fit}\left(X_{i}^{test}:\alpha^{test}\right)$$

where $X_{i}^{test}$ represents the set of experimental data used in the fitting using the GMM and $\alpha^{test}$ is the set of fitted parameters.

The criterion of cross validation quantifies a possible overfitting of the data while increasing the number of parameters and coefficients used in the model. The original set data are subdivided in *k* random samples. Each one of them is used in a validation set (called test), the data of the *k*-1 samples (train) are collected and fitted using the GMM model and the α^train^ variables are obtained. Then, using these parameters, the validation data (the values that were not used) are fitted and the MSE_i_^test^ of the validation set is calculated. This is iterated for all the N data points to average the cross validation MSE as a measure of the quality of the fitting.

Therefore, it was possible to define the number of Maxwell elements (arms) required in the GMM model. The order of the model is selected for the MSE to be minimal and the individual values of the fitting not to exceed an error of 10% (with an exception in Figure A8, where this threshold had to be increased to 20% because the total number of data of the relaxation curve was low (n = 70) and there was considerable noise on the data). The results of the cross validation were validated with the Akaike information criterion (AIC), as observed in Figure A7. This provides an estimation of the correctness and complexity of the model.

Globally, the best fitting order was for N = 4, as depicted by the lowest MSE (and AIC) value shown in Figure A7 below. However, we found that the error of each parameter individually increased with the number of degrees of freedom and it was decided to use a GMM fitting with N = 3, for which the parameters always presented an error lower than 10%.

## Appendix C. Validation and Comparison with Other Methods

**Table A1:** Pearson’s correlation between the Young’s modulus measured by microindentation (*E_H_*) and relaxation (*E_inst_* and *E_∞_*); in both cases a positive correlation was found indicating that if there is a change in the elastic behavior, both the Young’s modulus *E* and the long-term stiffness *E_∞_* will be affected in the same proportion since their correlation level is >0.9. On the other hand, with the instantaneous modulus *E_inst_* a correlation ~0.5 was obtained, which indicates a similar trend but not precisely in the same proportion. This effect is noted in Table 1 of the main text, where both the values of Young’s modulus and long-term stiffness of the 1 and 4 kPa samples measured in detergent maintained a 4-fold identical proportion between the expected values measured with a different technique (AFM). *r* (*E_H_*, *E_i_*) = Pearson correlation with E_H_.

	*E_H_*	*E_inst_*	*E_∞_*
*r* (*E_H_*, *E_i_*)	1.000	0.556	0.924
